# International students’ collective resilience in crisis: Sense of community reduced anxiety via social contact and social support during lockdown

**DOI:** 10.1016/j.heliyon.2023.e15298

**Published:** 2023-04-14

**Authors:** Xinyi Zhang, Alexander Scott English, Xiaoyuan Li, Yang Yang, Adrian Stanciu, Wang Shuang

**Affiliations:** a SISU Intercultural Institute, Shanghai International Studies University, China; b School of Psychology, Wenzhou-Kean University Wenzhou, Zhejiang China; c SISU Intercultural Institute, Shanghai International Studies University, Shanghai, China; d Leibniz Institute for the Social Sciences, Mannheim, Germany; e Department of Psychology, The Education University of Hongkong, Hong Kong

**Keywords:** International students, Sense of community, Anxiety, Social contact, Social support, COVID-19

## Abstract

**Objectives:**

The role of community in individuals' well-being has been extensively examined in the Western context. However, little is known about how the host community is related to sojourners' well-being in a crisis in an Asian context. The current study aims at exploring international students’ sense of community in the Chinese context under the direct threat of a global health crisis.

**Methods:**

Using a cross-sectional sample of 102 international students staying in Wuhan during the 76-day lockdown at the earliest stage of the COVID-19 pandemic, the current study explored the relationship between international students’ sense of community and anxiety, and the mediating role of social contact, social support from three key sources in the host community (host university, international students, and Chinese friends).

**Results:**

Results showed that participants’ stronger sense of community indirectly reduced anxiety via the role of sources of contact and support from the host community.

**Conclusions:**

This study provided further evidence to support the nurturance of the sense of community in community resilience and provided implications on how the host community can help to enhance sojourners’ psychological well-being in a global crisis.

## Public significance statement

Sense of community (SOC) was associated with mental health, especially in a crisis, but little evidence supports the benefits of SOC in sojourners' experience. This study showed that SOC improved international students' mental health in the COVID-19 pandemic in China and the importance to develop measures to promote international students’ engagement in the host community in a crisis.

## 1 Introduction

Host community plays a key role in benefiting the sojourner's acculturation process and mental health [[1], [2], [3]]. Community contact and support are essential for international students to manage crises and promote adjustment [4,5]. The COVID-19 pandemic has been a major crisis for global communities in the past few years and continues to disrupt people's life until today (e.g, the 2022 Shanghai lockdown [6]). International students, a sojourner group that encountered considerable challenges adapting to a new culture even before the pandemic [5,7,8], have been more susceptible to life disruptions during the pandemic compared to local populations [[9], [10], [11]]. It has been reported that international students encountered more mental disorders (e.g., anxiety, depression [12,13]), compared to previous times.

Although a wealth of studies addressed international students' mental health and coping strategies during the pandemic [10,14,15], scholars have paid scant attention to the role of perceived host community involvement and its relation to sojourners’ mental health outcomes during the pandemic, especially in China at the start of the outbreak. This study recruited a rare sample of international students who were unable to repatriate and experienced the 76-day Wuhan lockdown (from January 23rd to April 8th, 2020). This study aims to examine how the sense of community (SOC) might have been a positive psychological resource for international students in their response to the pandemic in Wuhan. Specifically, we sought to understand whether international students would report a SOC in the host community essential to their survival during lockdown, and if this SOC would alleviate their anxiety through social contact and social support during the lockdown.

### 1.1 Sense of community and mental health

Community can be broadly defined as a group of people with some shared elements ranging from geographical location to common interests or values [16]. One major type of community is the territorial community consisting of people with shared territorial or geographical locations [17]. SOC is the feeling of being part of a readily available, supportive, and dependable social structure [18], which is linked with the social environmental characteristics of a place [19]. It is established based on the sense of membership, the feeling that members matter to each other, and the belief that members’ needs will be fulfilled [20]. SOC is the basis for community members to feel, think and act on group terms, so as to build trust, maintain rules, and exchange support [20,21].

As suggested by the social identity approach, group membership is a basis to foster meaningful social interactions and to unlock the positive effects of social support on mental health [[22], [23], [24], [25]]. For example, Haslam and colleagues [26] found that perceived social support mediated the relationship between bomb disposer officers’ work-group identity and a lower level of work-related stress. Likewise, Frisch and colleagues [27] pointed out that social support reduces stress only when the support providers and support receivers formed a shared group identity. Shared group identity is part of the SOC as group identity demonstrates the membership aspect of SOC [16]. Therefore, the social identity approach implies that SOC can benefit individual mental health through social connections. It is well documented that SOC has a positive effect on mental health in various populations (e.g., college students, [28,29]; immigrants, [30]) and communities (e.g., the workplace, [31]; the neighborhood [32]). However, it is unclear if this benefit can be found among the sojourner groups in an international context [17].

### 1.2 Sense of community during a crisis

Although SOC is important to the quality of life and well-being in everyday life [17,33], it is especially significant in times of crisis in facilitating members to respond effectively to the crisis and fostering collective resilience [34,35]. Community is vital to individuals' survival and mental health in emergencies, as it provides essential resources (e.g., social ties and networks) for members to draw on [36]. SOC underpins these community ties when a crisis strikes [37,38]. When experiencing the same threatening event, members of a community tend to generate a sense of “togetherness” as they face a common fate and common goal [38,39]. In this way, the SOC forms a basis for social interaction and support in the community as members are more likely to be perceived as reliable sources of information, experience, knowledge, and support [40,41]. Thus, SOC is the determinant of social cohesion [42], community resilience and rebound in a crisis [35,43]. For example, previous studies identified SOC among survivors of several disasters or crises as well as the association between SOC and the perception or provision of support (e.g., the 2004 Beslan terrorist attack, [44]; the 2005 London bombing, [45]; the 2010 Chile earthquake, [37]; the 2015–2016 floods in York, UK [46]). The SOC as a key to handling crisis was particularly highlighted in the COVID-19 pandemic [25,47]. At the workplace, the SOC facilitated people's collaboration [48] and generated more positive perspectives toward the pandemic [49]. In the neighborhood, SOC predicted more altruistic behaviors among members during the pandemic [[50], [51], [52]].

### 1.3 International students and the host community

For international students staying far away from families and friends in a foreign culture, social contact and social support in the host community are paramount for them to acculturate in a new culture [1,53,54], and to cope with a crisis [5]. Previous research on international students and the host community highlighted the buffering effects of social support on the acculturation process. Specifically, researchers have identified key sources of contact and support stemming from the host community, including contact and support from the host networks [[55], [56], [57]] and from fellow international students in the host community [58,59], in alleviating international students’ stress and anxiety during the acculturation process. Furthermore, previous research suggests that host community support was more important in alleviating mental health problems among international students than other sources of support [[60], [61], [62]]. However, little is known about how international students perceive SOC when they are exposed to a public health emergency, and how the SOC is related to their mental health. In the current study, we examined the experiences of international students who were exposed to an unprecedented health threat and their SOC under this unusual circumstance.

### 1.4 The current study: international students in Wuhan lockdown

Wuhan was the first city in the world to undergo a strict lockdown to curb the COVID-19 pandemic, lasting for 76 days from January 23rd to April 8th, 2020 [63] (specific timeline of Wuhan lockdown can be found in supplementary material Figure S1). There was a high degree of uncertainty about the nature of the virus that troubled over 11 million residents in Wuhan and over 57 million residents in the entire Hubei province [64,65].

Understandably, staying in the epicenter at the start of the pandemic imposed more challenges on international students than those in other areas. Given the extreme uncertainty of this unusual “pneumonia” and being under lockdown, international students in Hubei reported more negative emotions (e.g., anxiety [66]), compared with their counterparts from other provinces [67], not only from worrying about getting infected but also due to unclear cultural expectations about what was happening around them [68].

International students from developing countries, who were the majority of participants in the current study, are a vulnerable population who have suffered more acculturative stress [69] and had limited mobility in the pandemic [70]. Apart from thousands of students and workers evacuated during the Wuhan outbreak, some countries, especially developing countries, were not able to evacuate their nationals in time [71]. For example, over 800 Pakistani students were stranded during Wuhan lockdown, yet the Pakistani ambassador to China informed students that they would not be evacuated due to the limited capacity of domestic health care infrastructure to handle the illness [72].

During the Wuhan lockdown, only essential workers (doctors, nurses, police) were allowed to go to work. Entrances of residential buildings (*xiaoqu*) were blocked to keep visitors from getting in and strictly guarded by security staff to keep insiders from getting out [73]. Only one person per household was allowed to go out every other day for a maximum of 30 min for essential supplies [74]. All students on-campus, including international students, were not allowed to leave their dormitory. Daily necessities were delivered to them by the university staff. One recent qualitative study interviewing international students in the Wuhan lockdown found that international students experienced increasing anxiety and other emotional challenges during the lockdown [68]. According to this study, these survivors felt an immediate sense of strong connection with the local community in Wuhan, and bonding with the university administration, international student community and local Chinese friends helped them to form a sense of community [68]. To date, no other study has empirically examined the collective experiences and social support of those international students in reducing anxiety.

In line with previous studies on SOC, we expected that the stronger SOC of international students was associated with less anxiety during the lockdown. Based on McMillan and Chavis' [20] theory of SOC, we specified the perceived community membership and perceived community involvement as the key elements constructing international students' SOC during the Wuhan lockdown. Although neighborhoods are traditionally seen as an indicator of territorial communities, the notion of community becomes increasingly broadened and fluid as mobility and globalization become prevalent in the past decades [75], which increases the complexity of the SOC [76]. With scant research exploring SOC for sojourners in a crisis, especially in an unprecedented threat spreading citywide and nationwide, we explore international students’ SOC through their recall of critical pandemic related events during the lockdown, instead of using scales which traditionally defined territorial community in the scope of a neighborhood [77,78].

Meanwhile, since the lockdown policy strictly restricted face-to-face interactions, individuals’ online contacts were paramount to fostering their social relationships with others. Qian and Hanser [73] pointed out that during the Wuhan lockdown, online social media (e.g., WeChat) were essential channels for citizens to get daily necessities and emotional support. Likewise, for international students during the lockdown, online platforms are primary means of contact with host universities and others in the community to gain information and support [68,79]. Therefore, in the current study, we expected online contact with the host community and the perceived social support from the host community would have a mediating effect on the relationship between SOC and anxiety. The core hypotheses in this study are:

Hypothesis 1 Participants' SOC would be associated with less anxiety.

Hypothesis 2 More frequent online contact within the community would be associated with more perceived social support from the community.

Hypothesis 3 Participants' SOC will have a negatively indirect effect on their anxiety through sources of online contact and sources of perceived social support.

## 2 Methods

### 2.1 Participants

Participants (*N* = 115; *M*_age_ = 29.28, *SD* = 4.43; age range = 20–41; 82 males and 33 females) were international students from 7 universities in Wuhan, China. 13 respondents were excluded due to incomplete answers or not being in Wuhan during the lockdown. The final sample consisted of 102 participants (*N* = 102; *M*_age_ = 29.39, *SD* = 4.37; age range = 21–41; 70 males and 32 females). Participants were first contacted by the fourth author who was an international staff support member in Wuhan prior to the pandemic. All participants were recruited via online WeChat groups given the strict social distancing policy. This study utilized snowball sampling. All participants provided a digital informed consent to participate and proceeded to complete the online survey. Participants were informed that their participation in the study was voluntary and they should feel free to leave at any time. Participants completed the online English survey in April 2020. Participants reported their length of stay in China (in months; *M* = 36.94, *SD* = 22.04) and their home country (Asia *n* = 80, 78.43%, Africa *n* = 14, 13.73%, and other regions *n* = 8, 7.84%). A full breakdown of demographic details can be found in Supplementary material Table S1.

### 2.2 Measures

#### 2.2.1 Sense of community (SOC)

##### 2.2.1.1 Critical event and emotion recall

The current study measured participants' SOC through their recall of critical events and emotional responses during the COVID-19 outbreak in Wuhan. Two open-ended questions in the survey asked participants to recall and then write down (1) the most challenging event during the pandemic and (2) what their feelings were towards this event. Participants’ SOC was coded based on the content of their answers.

##### 2.2.1.2 Coding protocol

**Fig. 1:**
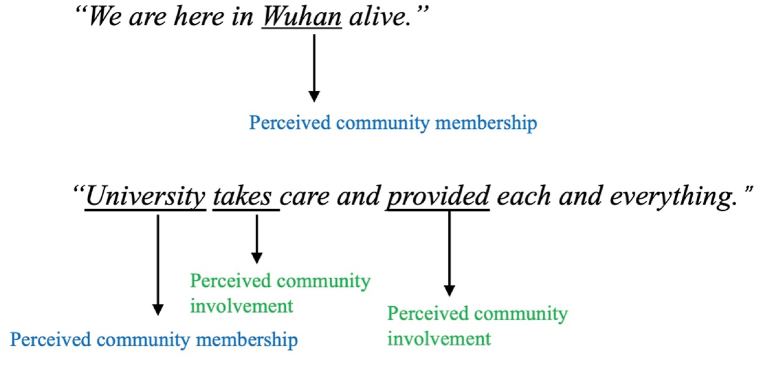
Examples of the SOC coding.

The coding protocol was developed based on a similar appraoch used in a recent study on emotional responses and coginitve process during the COVID-19 pandemic [80]. We coded nouns representing a community (e.g., university, Wuhan, China) as a major marker for “perceived community membership” with its following verbs coded as “perceived community involvement” (e.g., support, compensate) when it made reference to the behavior of the community within the same semantic context. Each code was given a point 1 value. Each participant's score of “sense of community” was calculated cumulatively based on the number of codes generated from the critical event and emotion recall. Examples are demonstrated in Fig. 1. More details of the coding protocol can be found in supplementary material 3.1.

##### 2.2.1.3 Inter-rater coding procedure

Given the small sample size, we used manual coding instead of computer-aided coding [81]. Without acknowledging the purpose of the study, three research assistants proficient in English were asked to code each participant's response according to the coding protocol. Before coding, the research assistants were trained by the first, second and third authors in two training sessions lasting 90 min each. In the first training session, the authors explained the coding rules to the three coders. After the first session, the coders independently coded the first 8 (7.84%) responses. In the second training session, the coders discussed the coding results with the authors to gain a better understanding of the coding protocol. After these two sessions, three coders coded the rest 94 responses independently. Inter-rater reliability was applied in this study to identify the reliability of open-ended survey analysis [82]. Inter-rater reliability test showed reliable coding agreement among the coders (κ > 0.95). The results presented here are based on an average score gained from the coding by three independent coders.

#### 2.2.2 Sources of contact and sources of support

Online social contact and social support were measured from major sources in the host community. After completing the COVID critical event and emotion recall task, participants were first asked to rate on an 11-point scale on the frequency of their contact online with three sources (1) host university faculty (*M* = 7.53, *SD* = 2.06), (2) Chinese friends (*M* = 8.02, *SD* = 1.85), and (3) other international students who also stayed (*M* = 6.85, *SD* = 2.46). Participants rated from −5 (much less) to 5 (much more) to indicate the frequency of contact compared to life before lockdown. The scores were recoded into a range from 0 to 10. Higher scores represented higher frequency of contact. Examples include “Compared to normal life prior to the Coronavirus outbreak, how frequently do you interact now with your university faculty online (social media, text, video)?". Cronbach's α value for these three sources of contact was 0.59.

Participants were then asked to rate on an 11-point scale to report their perceived social support from the same three sources as social contact (1) the host university (*M* = 8.32, *SD* = 2.01); (2) Chinese friends (*M* = 6.64, *SD* = 2.95); and (3) international students who stayed in China (*M* = 7.90, *SD* = 2.17). Participants rated from 0 (not helpful) to 10 (very helpful). An example of the survey questions is “Compared to normal life prior to the Coronavirus outbreak, how do you feel about the overall support from university faculty and administration?". Cronbach's α value for these three sources of support was 0.59.

#### 2.2.3 Anxiety

Participant's anxiety level was operationalized by four items from the sub-scale of Worries in the Perceived Stress Questionnaire [83], which “covers worries and anxious concerns for the future feelings of desperation and frustration” [84]. Participants were prompted to think about their situation in the past two weeks before they rated the items based on a 5-point Likert scale from “never” to “always”. Higher scores indicated a higher level of anxiety. Examples included “I am afraid of the future”, “I fear I may not manage to attain my goals” (α = 0.85). Further explanations can be found in Supplementary material SI-4.

### Ethical approval

This research was conducted in conformity with the ethical guidelines of the responsible institution and the Declaration of Helsinki. This research is affiliated with a larger project that received ethical approval from the Intercultural Institute at Shanghai International Studies University (Research Project Protocol # 2020-UNI-0211).

### 2.3 Analytical procedure

All analyses were done using SPSS and AMOS v.20 in the current study. Pearson correlation was used to perform correlation among major variables in SPSS. Thereafter, AMOS v.20 was used to construct a structural equation modeling (SEM) to test the predicted mediating role of sources of contact and support in the relationship between SOC and anxiety. A relatively good model-data fit was indicated by the following thresholds: a root mean square error of approximation (RMSEA) ≤ 0.06, a comparative fit index (CFI) ≥ 0.95 [85]. In addition, direct and indirect effects are estimated with bootstrapping (5000 iterations) with 95% bias-corrected confidence intervals. By repeated sampling with replacement from the original sample, bootstrapping is a non-parametric technique to generate a confidence interval. Details of factor loadings can be found in supplementary material 3.2.

Cohen's *d* [86] was applied to examine the effect size for the direct effects. The results showed that our sample was adequate to detect a small to medium effect size for the direct effect of SOC on sources of contact (Cohen's *d* = 0.14), sources of contact on sources of support (Cohen's *d* = 0.25), and sources of support on anxiety (Cohen's *d* = 0.30). Meanwhile, the Monte Carlo power analysis [87] was applied to examine the effect size for the indirect effects and the result also showed a medium effect size (power of 0.61) for the indirect effect of SOC on anxiety.

## 3 Results

### 3.1 Descriptive statistics

Table 1 provides an overview of descriptive analysis and correlations among the variables. As predicted under Hypothesis 1, international students’ SOC was associated with less anxiety (*r* = −0.22, *p* = 0.030). Meanwhile, international students SOC were positively linked with sources of contact (*r* = 0.25, *p* = 0.010) and support (*r* = 0.25, *p* = 0.010). Sources of contact was positively related to sources of support (*r* = 0.28, *p* = 0.005), and sources of support was negatively related to anxiety (*r* = −0.26, *p* = 0.009).

**Table 1:** Means, standard deviations and correlations of major variables.

*N* = 102	1	2	3	4	5	6	7	*M*	*SD*
1. Sense of community	–							0.17	0.54
2. Sources of contact	0.25**	–						7.47	1.59
3. Sources of support	0.25**	0.28**	–					7.62	1.78
4. Anxiety	−0.22*	0.01	−0.26**	–				2.80	1.14
5. Gender	0.14	0.12	0.16	−0.24*	–			0.69	0.47
6. Age	−0.04	−0.05	0.01	−0.11	0.23*	–		29.4	4.37
7. Education	−0.01	−0.05	0.13	−0.06	0.14	0.72***	–	2.54	0.78

***p ≤ 0.001, **p ≤ 0.01, *p ≤ 0.5.

Note. Sense of community is the averages core of “perceived community membership” and “perceived community involvement”; Gender: 1 = male, 0 = female; Education: 1 = Bachelor's degree, 2 = Master's degree, 3 = PhD, 4 = Postdoc.

### 3.2 Direct effect and indirect effect

**Fig. 2:**
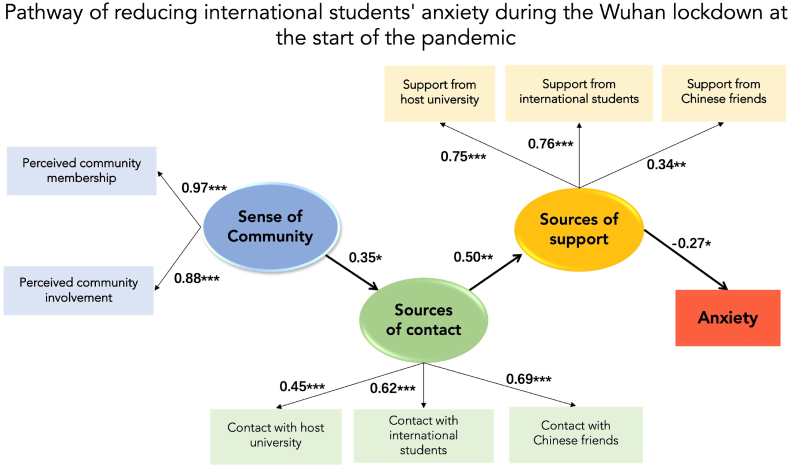
Model of SOC and anxiety. Note. *p < 0.05, **p < 0.01, ***p < 0.001. χ^2^ (25) = 1.10, *p* = 0.334, CFI = 0.989; RMSEA = 0.031. All reported estimates are standardized. The model with covariates can be found in supplementary material SI-6.

Results showed that SOC did not have a direct effect on anxiety (See supplementary material SI-7, Figure S3). Therefore, we established a new model without the direct effect of SOC on anxiety (Fig. 2). Results from the new model revealed that SOC was positively associated with sources of contact (*β* = 0.347, SE = 0.248, *p* = 0.013, *d* = 0.14). Sources of contact was positively associated with sources of support (*β* = 0.500, SE = 0.202, *p* = 0.003, *d* = 0.25), while sources of support were negatively associated with anxiety (*β* = −0.274, SE = 0.089, *p* = 0.020, *d* = 0.30). Results supported the hypothesis 2.

Meanwhile, SOC was indirectly and negatively associated with anxiety (*β* = −0.047, *p* < 0.001, 95% CI [−0.103, −0.017]). This result supported hypothesis 3. In addition, SOC was indirectly and positively associated with sources of support (*β* = 0.173, *p* < 0.001, 95% CI [0.071, 0.283]). Sources of contact were indirectly and negatively associated with anxiety (*β* = −0.137, *p* < 0.001, 95% CI [−0.240, −0.069]).

### 3.3 Robustness test and further analysis

Additional analyses were conducted to examine whether the results remained robust against a number of conditions, and in alternative models. We tested a) further breakdown between group analyses of participants’ location during the outbreak and their length of stay in China; b) if sources of contact predicted anxiety through SOC, and c) if sources of support predicted anxiety through SOC; d) if anxiety predicted SOC through social contact and support. No significant results were found in these analyses (see supplementary material SI-5, SI-7, SI-8, SI-9).

## 4 Discussion

This paper addresses how SOC plays a vital role in international students' mental health during the lockdown in Wuhan. Supporting our hypotheses 2 and 3, international students' SOC mitigated their anxiety through online social contacts and perceived social support from the host community. Results support the previous research that SOC was vital to the community and individual resilience in a crisis [21,35,40,88, 106]. The current study also extends the existing literature by using international students in Wuhan lockdown as a unique sample in an international context, highlighting the importance of online contact and perceived social support, and exploring SOC for sojourners in a crisis. The current study offers implications on how to promote international students’ engagement in the host community in helping them survive and recover from a crisis.

Although SOC did not directly predict anxiety, it had an indirect effect on anxiety via the mediating role of social contact and support from the host community. These findings are consistent with previous research that SOC is the key to promoting mental health in a crisis as it provides meaningful social connections, which become critical resources for individual resilience as well as community recovery during and after a crisis [38,88,89].

These findings extend existing research and offer practical implications in the following ways. First, this study extends previous research which focused on the SOC of same ethnicity groups in their home culture context [17,44,52]. Our findings suggest that the SOC is pivotal for sojourners to endure and recover from a major crisis as it activates positive social connections in the host community. Furthermore, this current study examined sojourners' SOC in a Chinese context. As far as we know, it is the first empirical study examining how international students from developing countries at the epicenter of the COVID-19 pandemic responded to this unprecedented challenge. Previous studies tend to examine individuals in China who coped with the pandemic in the community as an entire population without investigating the locally specific and group-specific perspectives [90,91]. However, international students in a host country may experience a crisis distinctively from host nationals given the unique challenges and stressors they were exposed to during the pandemic [[92], [93], [94]]. Surviving the pandemic can be especially challenging for international students when host community support has been inadequate as host members are also coping with the pandemic [70,93]. Findings in the current study also suggest that authorities in the host community (e.g., the university administration) should be perceived as part of the community to promote the community resilience in a crisis. These results offer practical implications to policymakers to develop measures to foster sojourners' sense of community and promote host community members’ involvement with international students, to facilitate effective responses to a crisis and alleviate mental health problems during and afterward a crisis.

Secondly, the current study highlights the importance of online channels or platforms for sojourners to gain social support and to maintain mental health, which offers implications for future intervention tools to support individuals recovering from the traumatic lockdown experience. As pointed out in one recent study on people who suffered during and after the pandemic, the online grief platform functioned as an effective intervention tool to enhance people's mental health [95]. This finding implies that governments and social organizations can develop more forms of online platforms to foster a sense of community, thereafter, to promote online contact and support to enhance people's mental health during and after COVID trauma.

Thirdly, the current study enhanced our understanding of sojourners' SOC in an urgent crisis in the host society by demonstrating SOC at multiple levels. Previous studies examined the SOC established among individuals without previous social bonds [45], as well as in pre-existing communities (e.g., the neighborhood [50]). In the current study, we found international students’ SOC was not homogeneously established in one geographic locale but in variant levels of communities (e.g., the host university, the host city, the host country). It can be explained as the SOC emerging in the pandemic could be fostered in a proximal neighborhood (e.g., the university) as well as a distal and broadened community (e.g., the city, the country), with the common goal and interests of surviving the pandemic. This finding echoes a socio-ecological perspective of resilience during the pandemic, which proposes cultivating resilience in the pandemic across multiple levels ranging from individual, organizational, community, to the national level [96].

## 5 Limitations and conclusion

While this study provides some contributions to the theory and underrepresented region in the world, there are some limitations that should be noted. First, the sample size is small even though this population is rare and elusive. Participants were composed of international students mainly from developing countries in Africa, South Asia and Latin America. While valuable in the sense of understanding SOC of individuals from the Global South, we have a limited number of participants in each country, thus we could not control the possible cross-cultural factors that might have influenced the participants' SOC. Meanwhile, this current study relies on cross-sectional data which limits our ability to analyze and understand the causal relationships between SOC and mental health [26]. Furthermore, we cannot discern the type of perceived social support in the host community. It is noteworthy that the participants' online contact with Chinese friends topped the contact frequency with university and fellow international students, yet the perceived support from Chinese friends was the lowest. It implies that certain sub-types of social support (e.g., emotional support) were not necessarily associated with contacts, but more relevant than other sub-types of support to mental health [97,98]. Future research should categorize sub-types of social support to capture the nuances. In addition, the current study measured SOC by coding the content of participants’ recall of pandemic related events. A lack of triangulation of research methods to cross-check the data restrained us from obtaining depth and gaining a thorough understanding of the phenomenon. At last, although this current study focused on the impact of SOC on mental health, it is noticeable that there are other factors (e.g., home environment, religion, and spirituality) important to mental health and resilience [99,100]. Future research can be conducted to examine the impact of home environment or religion.

In conclusion, the present study provides a novel perspective of SOC in a crisis, by focusing on international students during the lockdown in Wuhan at the beginning of COVID-19. Results of this study enhance our understanding of the power of SOC in influencing sojourners’ mental health in the host country in a crisis. It also highlights that the role of online contact and perceived social support in the relationship between SOC and individual psychology. It offers implications for future research on sojourners in crisis in an international context, as well as for policymakers to mobilize community resources to support the sojourner groups or other displaced people during a crisis and to recover after a crisis. The research is vital to understanding individuals' traumatic experiences after a life-changing event such as the COVID-19 lockdown, especially at the start when very little was known. This public health event might affect not only the current generation exposed to the trauma, but may continue to have an impact on the next generation [[101], [102]].

## Author contribution statement

Xinyi Zhang: Conceived and designed the experiments; Performed the experiments; Analyzed and interpreted the data; Contributed reagents, materials, analysis tools or data; Wrote the paper.

Alexander English: Conceived and designed the experiments; Performed the experiments; Analyzed and interpreted the data; Contributed reagents, materials, analysis tools or data.

Xiaoyuan Li: Conceived and designed the experiments; Contributed reagents, materials, analysis tools or data.

Yang Yang: Performed the experiments; Contributed reagents, materials, analysis tools or data.

Adrian Stanciu; Shuang Wang: Contributed reagents, materials, analysis tools or data.

Wang Shuang: Contributed reagents, materials, analysis tools or data.

**Funding**

This research did not receive any specific grant from funding agencies in the public, commercial, or not-for-profit sectors.

## Data availability statement

Data associated with this study has been deposited at https://osf.io/3vu7s/?view_only=7f6d527ee2dc4d76a4d0e523daf77a41.

## Declaration of competing interest

The authors declare that they have no known competing financial interests or personal relationships that could have appeared to influence the work reported in this paper.
