# Supplementary material for: International students’ collective resilience in crisis: Sense of community reduced anxiety via social contact and social support during lockdown
Source: Heliyon. 2023 Apr 14;9(4):e15298. doi: 10.1016/j.heliyon.2023.e15298 (PMC10130222; doi:10.1016/j.heliyon.2023.e15298)
Supplement: Multimedia component 1 [file mmc1.docx]

**Appendix A. Online Supplemental Materials**

In this supplementary material, we reported participants’ demographic details, timeline of the Wuhan lockdown, and details of major variables including the factor loadings. Meanwhile, we extended the analyses that tested our research hypotheses in the main text to address other potential predictors of international students’ anxiety, as well as alternative models.

**SI-1. Demographic details of participants in this study**

Table S1

| Country of citizenship | N | Percentage |
| --- | --- | --- |
| **Asia** | **80** | **78.43%** |
| 1. Pakistan | 70 | 68.63% |
| 1. Bangladesh | 2 | 1.96% |
| 1. Iran | 2 | 1.96% |
| 1. Yemen | 2 | 1.96% |
| 1. Afghanistan | 1 | 0.98% |
| 1. India | 1 | 0.98% |
| 1. Iraq | 1 | 0.98% |
| 1. Syria | 1 | 0.98% |
| **Africa** | **14** | **13.73%** |
| 1. Malawi | 3 | 2.94% |
| 1. Egypt | 2 | 1.96% |
| 1. Africa | 1 | 0.98% |
| 1. Angola | 1 | 0.98% |
| 1. Ethiopia | 1 | 0.98% |
| 1. Niger | 1 | 0.98% |
| 1. Nigeria | 1 | 0.98% |
| 1. Sierra Leone | 1 | 0.98% |
| 1. South Africa | 1 | 0.98% |
| 1. South Sudan | 1 | 0.98% |
| 1. Sudan | 1 | 0.98% |
| **Other regions** | **8** | **7.84%** |
| 1. Brazil | 1 | 0.98% |
| 1. Ecuador | 1 | 0.98% |
| 1. Grenada | 1 | 0.98% |
| 1. Samoa | 2 | 1.96% |
| 1. Spain 2. Prefer not to disclose | 1  2 | 0.98%  1.96% |
| Total | 102 | 100.00% |

Note: In SPSS “Prefer not to disclose” was coded as “other” for simplistic purposes but these two participants did not disclose their nationally.

**SI-2. Timeline of Covid-19 pandemic in Wuhan, China**


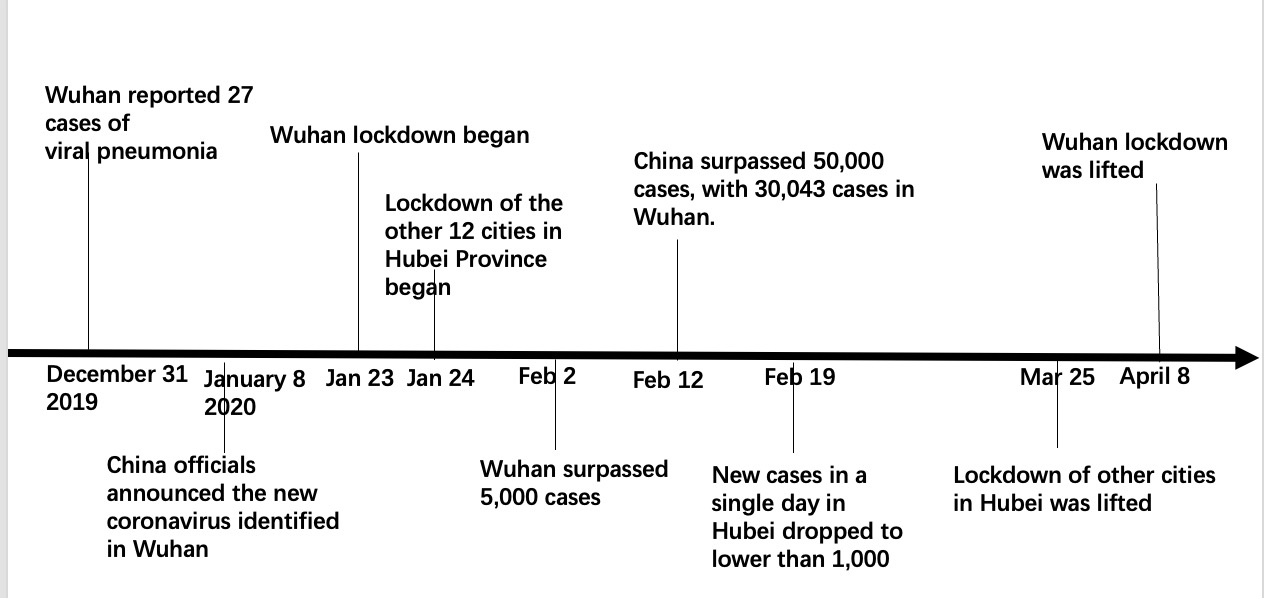
Figure S1. Timeline of important Covid-19 pandemic events in Wuhan

Source: National Health Commission of China and Wuhan Municipal Health Commission

**SI-3. Coding protocol and the factor loadings**

**3.1. Coding protocol**

Given the small sample size, we used manual coding instead of computer-aided coding [1]. In the current study, we specified the perceived community membership and perceived community involvement as the key elements constructing international students’ SOC during the Wuhan lockdown [2]. Firstly, the community membership was coded based on the notion of a place (e.g., the university, Wuhan city, China). Sense of place is regarded as part of the SOC [3], as people’s SOC is usually grounded in a sense of belonging to a particular location which gives meaning to people’s life [4,5]. This’s especially true during a disaster or crisis when the sense of place is the key to manifesting people’s sense of membership in a community [6,7]. Extracting components representing a location from a text to indicate the sense of place is widely used in place identity research [8,9]. Therefore, in this current study, we coded nouns of places in participants’ COVID event recall as a marker for perceived community membership.

Secondly, we coded community actions (e.g., compensate, support, take care) as the perceived community involvement. These actions indicated that from the participants’ perspective, what the host community had done during the lockdown.

**3.2 Factor loadings**

Factor loadings results of SOC, sources of support, and sources of contact are presented in Table S2

Table S2 Factor loading for major latent variables in the study

| Items | Estimate | *SE* | *p* |
| --- | --- | --- | --- |
| **Sense of community** |  |  |  |
| Perceived community membership | 0.98 | - | - |
| Perceived community involvement | 0.87 | 0.10 | <.001 |
| **Sources of support** |  |  |  |
| Support from university | 0.73 | - | - |
| Support from international students | 0.78 | 0.31 | <.001 |
| Support from Chinese friends | 0.34 | 0.26 | <.01 |
| **Sources of contact** |  |  |  |
| Contact with university | 0.48 | 0.19 | <.001 |
| Contact with international students | 0.58 | 0.39 | <.01 |
| Contact with Chinese friends | 0.74 | - | - |

Note: All reported estimates are standardized. A constraint was set on one indicator in each latent variable, which means the unstandardized path from the latent factor to the indicator was fixed to 1, therefore there is no *SE* for that path.

Furthermore, noticing that the SOC is relatively low (M=0.17, SD=0.54), we coded the alternative focus—sense of self, using the same method as coding SOC in the current study. The sense of self was coded as the perception of self (represented by the pronoun “I”) and the behavior of self (*M*= 1.37, *SD*= 2.83). Three coders were trained and then coded independently, thereafter reaching a satisfactory agreement (κ > .90). Results of factor loadings is presented in Table S3.

Table S3 factor loadings for sense of self and other latent variables in the study

| Items | Estimate | *SE* | *p* |
| --- | --- | --- | --- |
| **Sense of self** |  |  |  |
| Simple description of self | 0.97 | - | - |
| Behavior of self | 0.90 | 0.08 | <.001 |
| **Sense of community** |  |  |  |
| Perceived community membership | 0.98 | - | - |
| Perceived community involvement | 0.87 | 0.07 | <.001 |
| **Sources of support** |  |  |  |
| Support from university | 0.97 | - | - |
| Support from international students | 0.58 | 0.23 | <.01 |
| Support from Chinese friends | 0.23 | 0.20 | 0.89 |
| **Sources of contact** |  |  |  |
| Contact with university | 0.50 | 0.19 | <.001 |
| Contact with international students | 0.56 | 0.33 | <.01 |
| Contact with Chinese friends | 0.75 | - | - |

Note: All reported estimates are standardized. A constraint was set on one indicator in each latent variable, which means the unstandardized path from the latent factor to the indicator was fixed to 1, therefore there is no *SE* for that path.

**SI-4. Measurement of anxiety**

Theories and studies in the literature tend to conceptualize worry as a subtype of anxiety[10,11], and as an indicator of poor mental health [12]. Scales of worry and anxiety are sometimes used interchangeably [13]. Worry was often measured as a form of anxiety or as a construct closely associated with it [14]. Moreover, worry is related to fear and uncertainty about the future [15], which fits the current study examining the earliest stages of the COVID-19 pandemic when individuals had a high level of uncertainty [16]. Therefore, in the current study, the participants’ anxiety was measured by a subscale of worries extracted from the Perceived Stress Questionnaire [17], which “covers worries and anxious concerns for the future feelings of desperation and frustration” [18]. This subscale includes four items as followed: “I am afraid of the future”, “I fear I may not manage to attain my goals”, “I am afraid for the future”, “I feel under pressure from deadlines”. Participants rated based on a 5-point Likert scale from “never” to “always” (α = .85, *M* = 2.80, *SD* = 1.14).

**SI-5. Possible covariates in the current study**

**5.1 Did location during the outbreak make a difference?**

The place international students stay might have an impact on their mental health. International students living in dormitories in China during the COVID-19 pandemic reported more anxiety than those staying outside campus [19]. We conducted an ANOVA test to examine the effect of location differences on major variables.

These results suggested that staying at the campus or not has an impact on participants’ contact with the university and support from Chinese friends (more contact with the university when staying at campus, and more support from local Chinese friends when staying outside campus). However, there was no significant difference regarding other sources of contact or support, the SOC, and anxiety (see Table S4).

Table S4. location comparison of major variables

|  |  | **Sense of community** | | **Sources of support** | | | | **Sources of contact** | | | **Anxiety** | |
| --- | --- | --- | --- | --- | --- | --- | --- | --- | --- | --- | --- | --- |
|  | n | Perceived community membership | Perceived community involvement | University | International students | Chinese friends | University | | International students | Chinese friends | | Anxiety |
|  |  | M(SD) | M(SD) | M(SD) | M(SD) | M(SD) | M(SD) | | M(SD) | M(SD) | | M(SD) |
| **Staying at Campus** | 57 | 0.29(0.78) | 0.11(0.44) | 8.40(1.85) | 7.95(1.94) | 5.88(2.92) | 7.93(1.66) | | 6.88(2.70) | 8.20(1.74) | | 2.82(1.15) |
| **Staying outside Campus** | 45 | 0.21(0.71) | 0.05(0.30) | 8.24(2.22) | 7.84(2.45) | 7.60(2.73) | 7.02(2.40) | | 6.82(2.16) | 7.80(1.98) | | 2.78(1.14) |
| *F-tests* |  | F(1,100)=0.23  P=0.63 | F(2,101)=0.49  P=0.49 | F(1,100)= 0.12  P=0.73 | F(1,100)=0.06  P=0.81 | F(1,100)= 9.30 P=0.003 | F(1,100)= 5.10  P=0.027 | | F(1,100)= 0.01  P=0.91 | F(1,100)=1.14  P=.29 | | F(1,100)= 0.03 P=0.87 |

**5.2. Did length of stay in China make a difference?**

Length of stay is an important predictor of sojourners’ acculturation outcomes in the host society [20,21] and a factor influencing the sense of community [22]. Rasmi and colleagues [23] pointed out that international students had more connections with the host country members after the length of stay of 18 months. Alam and colleagues [19] reported that international students staying in China for more than two years faced a higher risk of psychological outcomes (e.g., anxiety, depression, fear). Participants’ length of stay in the current study ranged from 8 months to 104 months (*M* = 36.94, *SD* = 22.04). To examine the impact of short-term and long-term length of stay in China, we created a categorical variable: short-term residents (i.e., length of stay is less than 3 years; n = 57) vs. long-term residents (i.e., length of stay is longer than 3 years; n = 45). The ANOVA test results (Table S5) revealed a slight difference in the frequency of contact with the host university in terms of length of stay. However, there was no significant difference in length of stay in SOC, sources of support, other sources of contact, and anxiety.

Table S5 Comparison of length of stay among major variables

|  |  | **Sense of community** | | **Sources of support** | | | **Sources of contact** | | | **Anxiety** | |  |
| --- | --- | --- | --- | --- | --- | --- | --- | --- | --- | --- | --- | --- |
|  | n | Perceived community membership | Perceived community involvement | University | International students | Chinese friends | University | International students | Chinese friends | | Anxiety | |
|  |  | M(SD) | M(SD) | M(SD) | M(SD) | M(SD) | M(SD) | M(SD) | M(SD) | | M(SD) | |
| **Short-term** | 57 | 0.34(0.92) | 0.13(0.50) | 8.44(1.99) | 7.68(2.32) | 6.58(2.96) | 7.89(2.11) | 6.82(2.44) | 8.05(1.93) | | 2.80(1.16) | |
| **Long-term** | 45 | 0.15(0.41) | 0.02(0.11) | 8.18(2.05) | 8.18(1.94) | 6.71(2.96) | 7.07(1.92) | 6.89(2.51) | 7.98(1.76) | | 2.81(1.13) | |
| *F-tests* |  | F(1,100)=1.67  P=0.20 | F(2,101)=1.97  P=0.16 | F(1,100)= 0.42  P=0.52 | F(1,100)=1.31  P=0.26 | F(1,100)= 0.50 P=0.82 | F(1,100)= 4.12  P=0.04 | F(1,100)= 0.02  P=0.90 | F(1,100)=0.41  P=0.84 | | F(1,100)= 0.003 P=0.96 | |

**SI-6. The model with covariates**

Concerning the small sample size in the current study, we tested the hypothesized model in the main text without controlling other confounding variables (see the main text Figure3). In this supplementary material, we represented the hypothesized model with controlling for age, gender, education level, and length of stay.


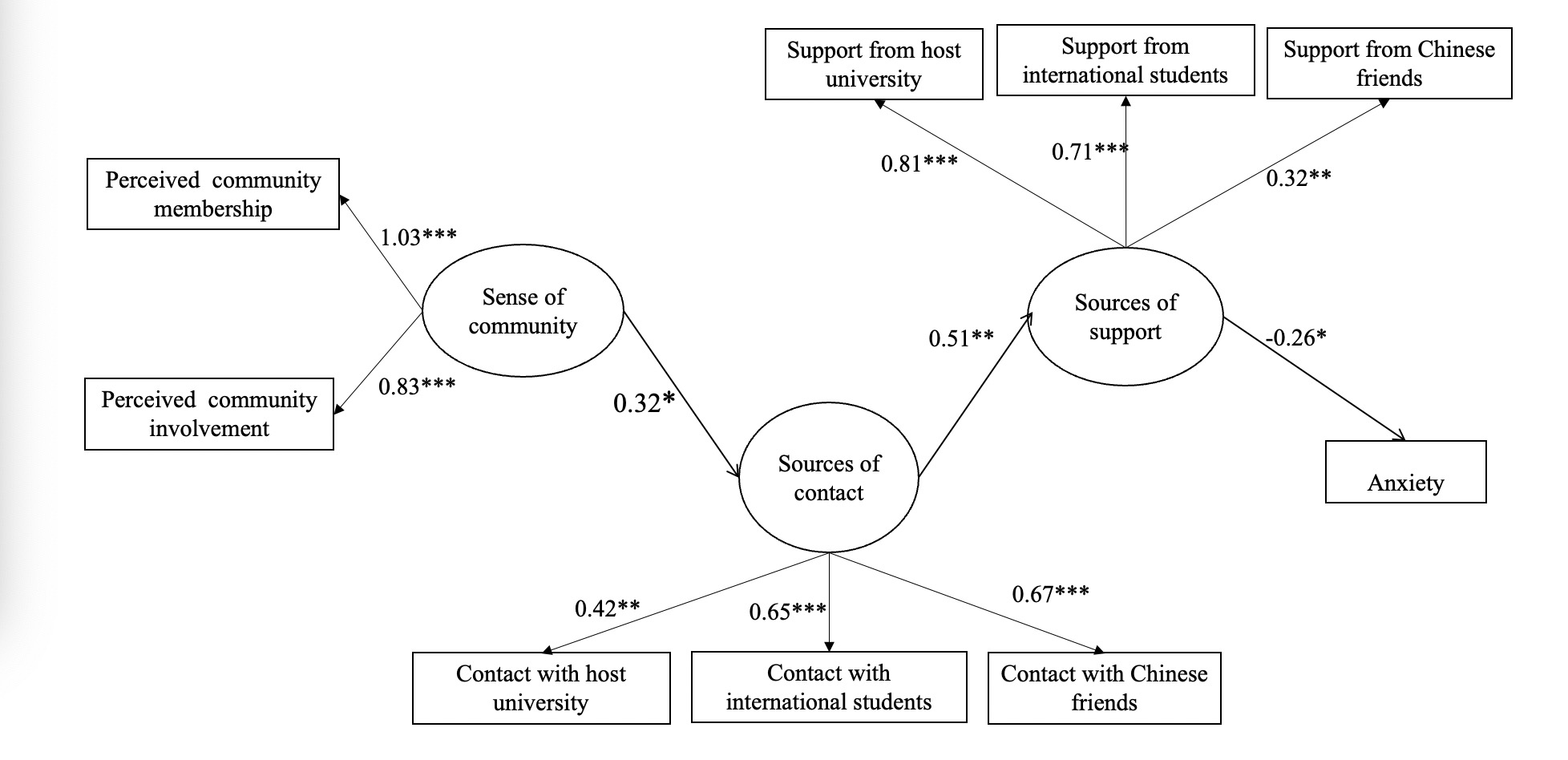


Figure S2. χ^2^(45) = 1.07, *p* = 0.340, CFI = 0.990, RMSEA = 0.027. All reported estimates are standardized. * *p* < 0.05, ** *p* < 0.01, *** *p* < 0.001. Age, gender, education level and length of stay were controlled.

**Direct effects**: Results revealed that SOC positively predicted sources of contact (*β* = 0.317, SE = 0.227, *p* = 0.023). Sources of contact positively predicted sources of support (*β* = 0.511, SE = 0.215, *p* = 0.002), sources of support negatively predicted anxiety (*β* = -0.263, SE = 0.085, *p* = 0.029). **Indirect effects**: SOC was indirectly and negatively associated with anxiety (*β* = −0.043, *p* < 0.001, 95% CI [-0.105, -0.013]). SOC was indirectly and positively associated with support (*β* = 0.162, *p* < 0.001, 95% CI [0.060, 0.284]). Sources of contact was indirectly and negatively associated with anxiety (*β* = −0.135, *p* < 0.001, 95% CI [-0.246, -0.064]).

**SI-7. The model with a direct effect of SOC on anxiety**

We hypothesized that participants’ SOC directly predict anxiety. However, the result showed an insignificant effect of SOC on anxiety (Figure. S3)

**
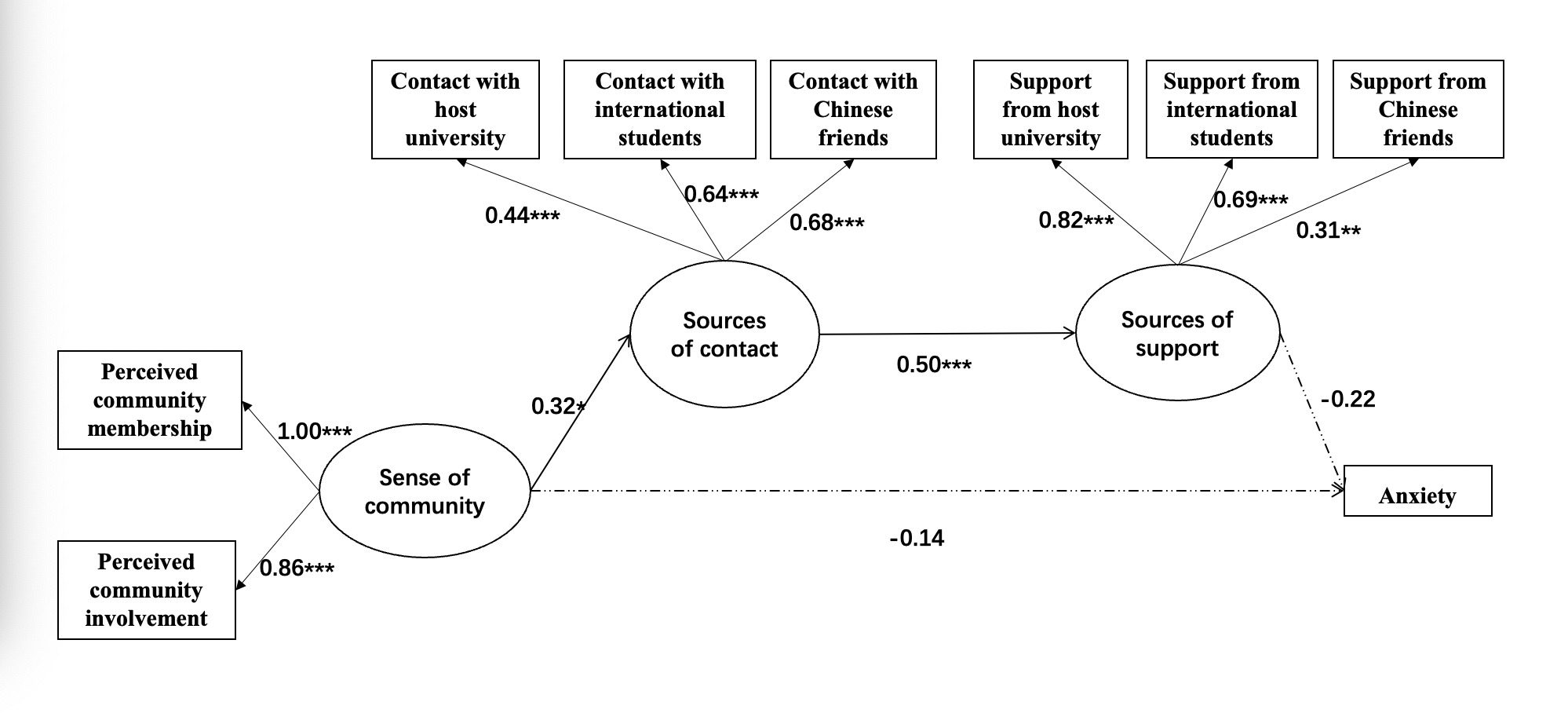
**

Figure S3. χ^2^(44) = 1.06, *p* = 0.368, CFI = 0.992, RMSEA = 0.024). * *p* < 0.05, ** *p* < 0.01, *** *p* < 0.001. Age, gender, education level and length of stay were controlled.

**SI-8. Models with alternative paths: did sources of contact predict less anxiety through sources of support and SOC?**

Although the literature explicated that in a crisis the SOC benefited wellbeing through social interactions and sources of support [24–26], it’s also possible that increasing contacts and support can foster a stronger SOC [27,28]. Therefore, we tested if sources of contact predicted less anxiety through sources of support and SOC. The results showed an insignificant path in the alternative models.


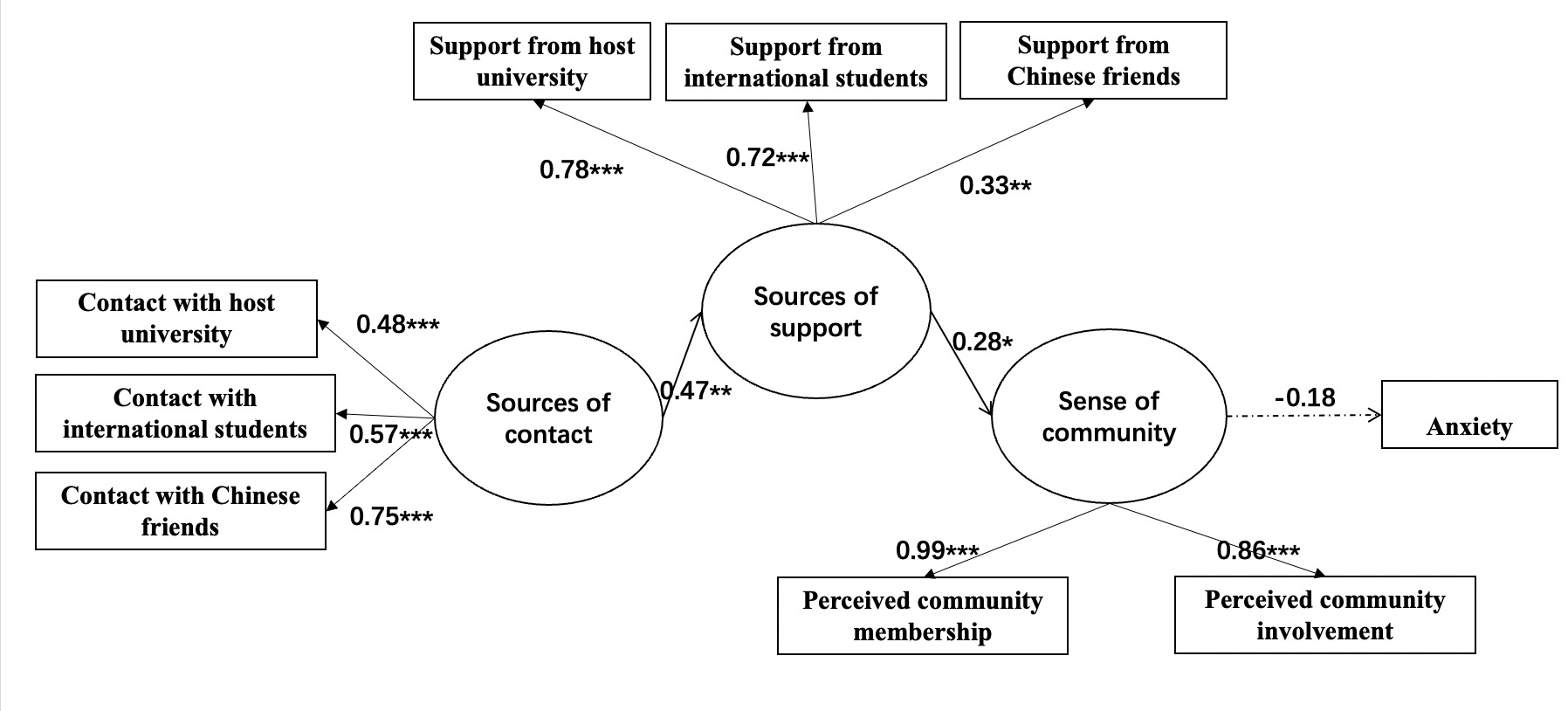


Figure S4. (χ2(45) = 1.16, p = 0.214, CFI = 0.978, RMSEA = 0.040. * p < 0.05, ** p < 0.01, *** p < 0.001. Age, gender, education level and length of stay were controlled.

As showed in Figure S4, sources of contact predicted higher SOC through sources of support. However, SOC did not predict anxiety, meanwhile sources of contact did not indirectly impact anxiety through sources of support and SOC. These results implies that more frequent contact could lead to more SOC; however, contacts or support couldn’t predict anxiety through SOC.

Meanwhile, we also tested if sources of contact predicted support and anxiety through SOC. Results are showed in Figure S5.


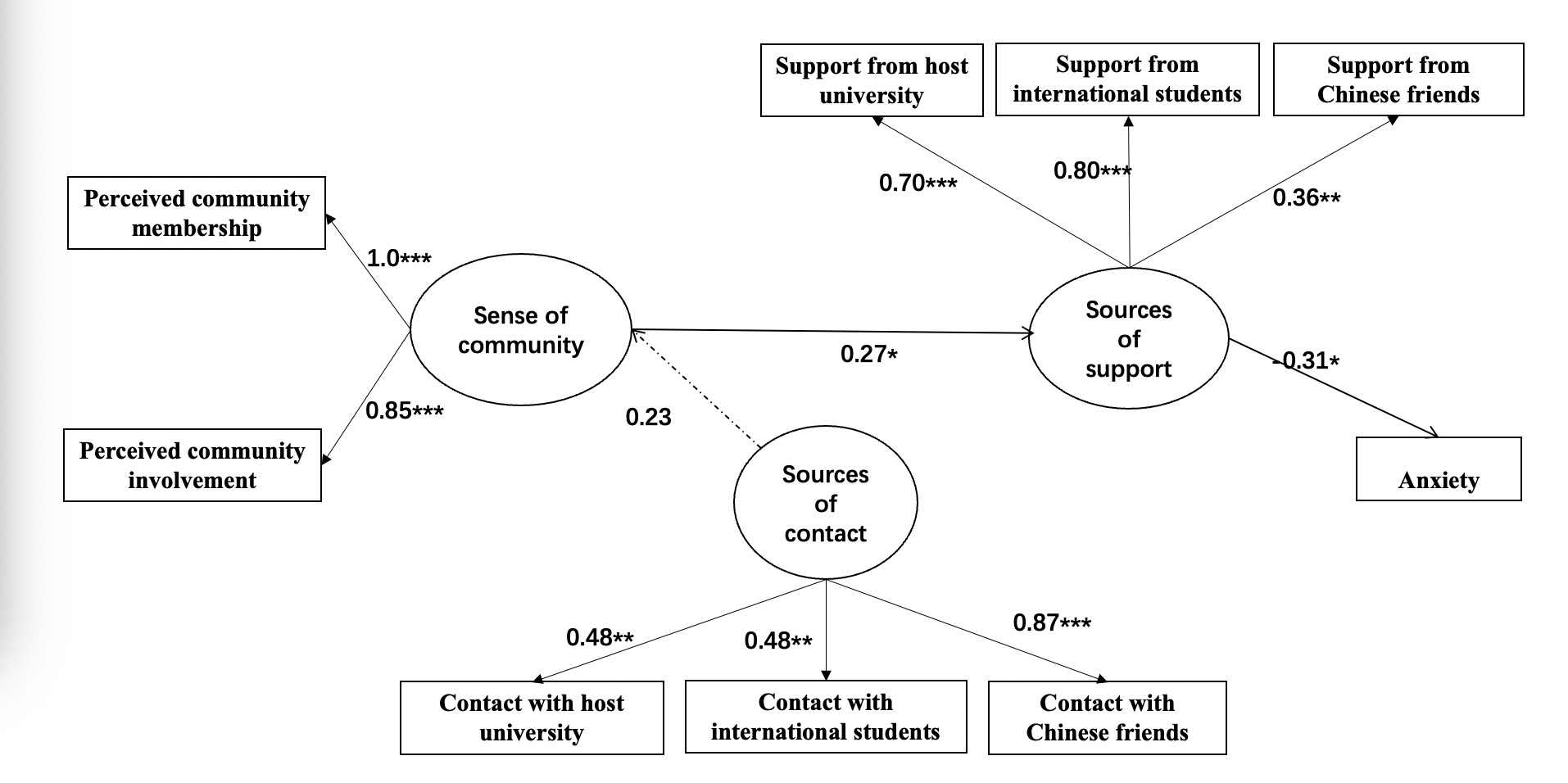


Figure S5. χ^2^(45) = 1.19, *p* = 0.182, CFI = 0.974, RMSEA = 0.043). * *p* < 0.05, ** *p* < 0.01, *** *p* < 0.001. Age, gender, education level and length of stay were controlled.

Figure S5. revealed that sources of contact did not directly predict SOC. Sources of contact did not have an impact on anxiety through SOC and sources of support.

**SI-9. Models with alternative paths: did anxiety predict less SOC through contact and support?**

We also tested if it’s international students’ anxiety would be associated with SOC through social contact and support. As showed in Figure S6, anxiety didn’t predict social contact. Therefore, anxiety wasn’t associated with SOC through social contact and support.


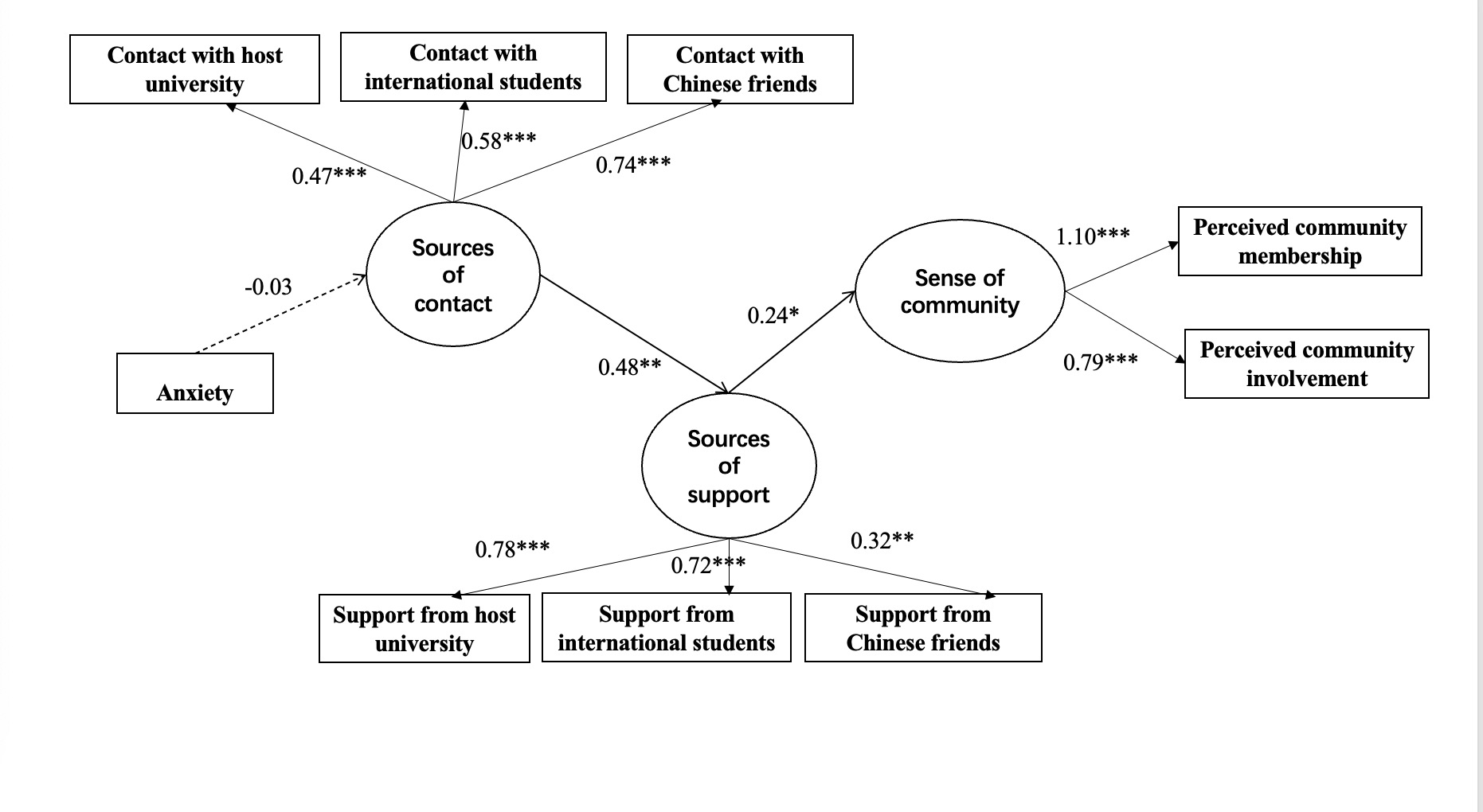


Figure S6. χ^2^(45) = 1.225, *p* = 0.144, CFI = 0.969, RMSEA = 0.047). * *p* < 0.05, ** *p* < 0.01, *** *p* < 0.001. Age, gender, education level and length of stay were controlled.

References

[1] Nshom E, Khalimzoda I, Sadaf S, Shaymardanov M. Perceived threat or perceived benefit? Immigrants’ perception of how Finns tend to perceive them. International Journal of Intercultural Relations 2022;86:46–55. https://doi.org/10.1016/j.ijintrel.2021.11.001.

[2] McMillan DW, Chavis DM. Sense of community: A definition and theory. J Community Psychol 1986;14:6–23. https://doi.org/10.1002/1520-6629(198601)14:1<6::AID-JCOP2290140103>3.0.CO;2-I.

[3] DeYoung SE, Peters M. My Community, My Preparedness: The Role of Sense of Place, Community, and Confidence in Government in Disaster Readiness. International Journal of Mass Emergencies & Disasters 2016;34:250–82. https://doi.org/10.1177/028072701603400204.

[4] Proshansky HM, Fabian AK, Kaminoff R. Place-identity: Physical world socialization of the self. Journal of Environmental Psychology 1983;3:57–83. https://doi.org/10.1016/S0272-4944(83)80021-8.

[5] Puddifoot JE. Exploring ?personal? and ?shared? sense of community identity in Durham City, England. J Community Psychol 2003;31:87–106. https://doi.org/10.1002/jcop.10039.

[6] Chamlee-Wright E, Storr VH. “There’s No Place like New Orleans”: Sense of Place and Community Recovery in the Ninth Ward after Hurricane Katrina. Journal of Urban Affairs 2009;31:615–34. https://doi.org/10.1111/j.1467-9906.2009.00479.x.

[7] Dueck A, Byron K. Community, Spiritual Traditions, and Disasters in Collective Societies. Journal of Psychology and Theology 2011;39:244–54. https://doi.org/10.1177/009164711103900307.

[8] Brace C. Finding England everywhere: regional identity and the construction of national identity, 1890-1940. Ecumene 1999;6:90–109. https://doi.org/10.1177/096746089900600105.

[9] Peng J, Strijker D, Wu Q. Place Identity: How Far Have We Come in Exploring Its Meanings? Front Psychol 2020;11:294. https://doi.org/10.3389/fpsyg.2020.00294.

[10] Borkovec TD, Robinson E, Pruzinsky T, DePree JA. Preliminary exploration of worry: Some characteristics and processes. Behaviour Research and Therapy 1983;21:9–16. https://doi.org/10.1016/0005-7967(83)90121-3.

[11] Spielberger CD. Test Anxiety Inventory. The Corsini Encyclopedia of Psychology, 2010, p. 1–1. https://doi.org/10.1002/9780470479216.corpsy0985.

[12] Eysenck MW. Anxiety: The Cognitive Perspective. London: Psychology Press; 1992. https://doi.org/10.4324/9780203775677.

[13] Wisocki PA, Handen B, Morse CK. The Worry Scale as a measure of anxiety among homebound and community active elderly. The Behavior Therapist 1986;9:91–5.

[14] Boehnke K, Schwartz S, Stromberg C, Sagiv L. The Structure and Dynamics of Worry: Theory, Measurement, and Cross-National Replications. Journal of Personality 1998;66:745–82. https://doi.org/10.1111/1467-6494.00031.

[15] MacLeod AK, Williams JM, Bekerian DA. Worry is reasonable: The role of explanations in pessimism about future personal events. Journal of Abnormal Psychology 1991;100:478–86. https://doi.org/10.1037/0021-843X.100.4.478.

[16] Zhuo L, Wu Q, Le H, Li H, Zheng L, Ma G, et al. COVID-19-Related Intolerance of Uncertainty and Mental Health among Back-To-School Students in Wuhan: The Moderation Effect of Social Support. IJERPH 2021;18:981. https://doi.org/10.3390/ijerph18030981.

[17] Levenstein S, Prantera C, Varvo V, Scribano ML, Berto E, Luzi C, et al. Development of the perceived stress questionnaire: A new tool for psychosomatic research. Journal of Psychosomatic Research 1993;37:19–32. https://doi.org/10.1016/0022-3999(93)90120-5.

[18] Fliege H, Rose M, Arck P, Walter OB, Kocalevent R-D, Weber C, et al. The Perceived Stress Questionnaire (PSQ) Reconsidered: Validation and Reference Values From Different Clinical and Healthy Adult Samples: Psychosomatic Medicine 2005;67:78–88. https://doi.org/10.1097/01.psy.0000151491.80178.78.

[19] Alam MdD, Lu J, Ni L, Hu S, Xu Y. Psychological Outcomes and Associated Factors Among the International Students Living in China During the COVID-19 Pandemic. Front Psychiatry 2021;12:707342. https://doi.org/10.3389/fpsyt.2021.707342.

[20] Berry JW. Stress perspectives on acculturation. In: Sam DL, Berry JW, editors. The Cambridge Handbook of Acculturation Psychology. 1st ed., Cambridge University Press; 2006, p. 43–57. https://doi.org/10.1017/CBO9780511489891.007.

[21] Ward C, Bochner S, Furnham A. Psychology Culture Shock. London: Routledge; 2020. https://doi.org/10.4324/9781003070696.

[22] Helliwell J, Barrington-Leigh C. How much is social capital worth?. The social cure, Psychology Press; 2012, p. 55–71. https://doi.org/10.4324/9780203813195.

[23] Rasmi S, Safdar S, Lewis J. A longitudinal examination of the MIDA model with international students, 2010, p. 42–57.

[24] Kaniasty K. Social support, interpersonal, and community dynamics following disasters caused by natural hazards. Current Opinion in Psychology 2020;32:105–9. https://doi.org/10.1016/j.copsyc.2019.07.026.

[25] Moscardino U, Scrimin S, Capello F, Altoè G. Social support, sense of community, collectivistic values, and depressive symptoms in adolescent survivors of the 2004 Beslan terrorist attack. Social Science & Medicine 2010;70:27–34. https://doi.org/10.1016/j.socscimed.2009.09.035.

[26] Stevenson C, Wakefield JRH, Felsner I, Drury J, Costa S. Collectively coping with coronavirus: Local community identification predicts giving support and lockdown adherence during the COVID‐19 pandemic. Br J Soc Psychol 2021;60:1403–18. https://doi.org/10.1111/bjso.12457.

[27] Mak WWS, Cheung RYM, Law LSC. Sense of Community in Hong Kong: Relations with Community-Level Characteristics and Residents’ Well-Being. American Journal of Community Psychology 2009;44:80–92. https://doi.org/10.1007/s10464-009-9242-z.

[28] Paton D, Irons M. Communication, Sense of Community, and Disaster Recovery: A Facebook Case Study. Front Commun 2016;1. https://doi.org/10.3389/fcomm.2016.00004.

**Appendix B.** **Experience of international students in Wuhan during the COVID-19**^^[[1]](#footnote-1)^^

1. Country of citizenship:

_________________________________

2. Gender [单选题]

| ○Male |
| --- |
| ○Female |

3. The year you born [填空题]

_________________________________

4. When did you start to study in China? [填空题]

_________________________________

5. What is your university name? [填空题]

You could use the abbreviation.

_________________________________

6. What degree are currently you studying now? [单选题]

| ○Bachelor's degree |
| --- |
| ○Master's degree |
| ○PhD |
| ○Posdoc |
| ○Language student |

7. Where did you stay during the outbreak? (Jan 20-Now)? [填空题]

_________________________________

8. When did you get the notification of the outbreak? (please write down the date) [填空题] *

If you don't remember the date, write down "0".

_________________________________

9. How much has the Coronavirus impacted you? [单选题] *

| Not at all | ○1 | ○2 | ○3 | ○4 | ○5 | ○6 | ○7 | ○8 | ○9 | ○10 | A lot |
| --- | --- | --- | --- | --- | --- | --- | --- | --- | --- | --- | --- |

10. At the **beginning** of the outbreak, how often did the university check on you? [单选题] *

| ○4 times a day |
| --- |
| ○3 times a day |
| ○2 times a day |
| ○1 time a day |
| ○Every two days |
| ○Other _________________ * |

11. At the **beginning** of the outbreak, how comfortable do you feel with university’s checking?

Please slide.

[输入0(very uncomfortable)到100(very comfortable)的数字]*

________________________________

12. **Currently**, how often does university check on you since the Coronavirus outbreak? [单选题] *

| ○4 times a day |
| --- |
| ○3 times a day |
| ○2 times a day |
| ○1 time a day |
| ○Every two days |
| ○Other _________________ * |

13. Currently, how comfortable do you feel with university’s checking?

Please slide.

[输入0(very uncomfortable)到100(very comfortable)的数字]*

________________________________

You are invited to think about **the most challenging social issue** you have encountered since the outbreak. Please spend some time thinking how it has affected you personally.

14. When did you first encounter this social issue? [单选题] *

| ○Last couple of days |
| --- |
| ○This week |
| ○Several weeks ago |
| ○More than a few weeks ago |

15. This social issue happened in which day of that week? [单选题] *

| ○Monday |
| --- |
| ○Tuesday |
| ○Wednesday |
| ○Thursday |
| ○Friday |
| ○Saturday |
| ○Sunday |
| ○Don't remember |

16. Were there other people involved in this issue? [单选题] *

| ○Yes |
| --- |
| ○No |

17. Please describe this issue in the box below [填空题] *

Anything you want to talk about.

_________________________________

18. As you are thinking about this issue now, what thoughts and emotions come to your mind? [填空题] *

Please describe your feelings when you think of it now.

_________________________________

19.

While I have been reflecting on this issue, I……….

[矩阵单选题] *

|  | Not at all | Slightly | Somewhat | Moderately | Very much |
| --- | --- | --- | --- | --- | --- |
| Considered the perspectives of the other people involved | ○ | ○ | ○ | ○ | ○ |
| Took time to consider other people's interests before coming to a conclusion | ○ | ○ | ○ | ○ | ○ |
| Thought the issue could unfold in many different ways | ○ | ○ | ○ | ○ | ○ |
| Looked for different available solutions | ○ | ○ | ○ | ○ | ○ |
| Double-checked whether my opinion on the issue might be incorrect | ○ | ○ | ○ | ○ | ○ |

| Looked for additional information before forming my opinion | ○ | ○ | ○ | ○ | ○ |
| --- | --- | --- | --- | --- | --- |
| Considered first whether a compromise was possible | ○ | ○ | ○ | ○ | ○ |
| Tried my best to find a way to accommodate interests of all people involved | ○ | ○ | ○ | ○ | ○ |
| Checked the reliability of my sources before forming my opinion | ○ | ○ | ○ | ○ | ○ |
| Considered where my information on the issue is coming from | ○ | ○ | ○ | ○ | ○ |

**Compared** to normal life prior to the Coronavirus, **how frequently** do you...?^[[2]](#footnote-2)^2

21. interact with your university faculty online (social media, text, video games)?

"0" means the frequency is the same as before.

| Much less | ○-5 | ○-4 | ○-3 | ○-2 | ○-1 | ○0 | ○1 | ○2 | ○3 | ○4 | ○5 | Much more |
| --- | --- | --- | --- | --- | --- | --- | --- | --- | --- | --- | --- | --- |

22. interact with your university faculty face to face?

"0" means the frequency is the same as before.

| Much less | ○-5 | ○-4 | ○-3 | ○-2 | ○-1 | ○0 | ○1 | ○2 | ○3 | ○4 | ○5 | Much more |
| --- | --- | --- | --- | --- | --- | --- | --- | --- | --- | --- | --- | --- |

23. interact with your family online?

"0" means the frequency is the same as before.

| Much less | ○-5 | ○-4 | ○-3 | ○-2 | ○-1 | ○0 | ○1 | ○2 | ○3 | ○4 | ○5 | Much more |
| --- | --- | --- | --- | --- | --- | --- | --- | --- | --- | --- | --- | --- |

24. interact with your friends face to face?

"0" means the frequency is the same as before.

| Much less | ○-5 | ○-4 | ○-3 | ○-2 | ○-1 | ○0 | ○1 | ○2 | ○3 | ○4 | ○5 | Much more |
| --- | --- | --- | --- | --- | --- | --- | --- | --- | --- | --- | --- | --- |

25. interact with your friends online?

"0" means the frequency is the same as before.

| Much less | ○-5 | ○-4 | ○-3 | ○-2 | ○-1 | ○0 | ○1 | ○2 | ○3 | ○4 | ○5 | Much more |
| --- | --- | --- | --- | --- | --- | --- | --- | --- | --- | --- | --- | --- |

26. interact online with other international students who also stayed?

"0" means the frequency is the same as before.

| Much less | ○-5 | ○-4 | ○-3 | ○-2 | ○-1 | ○0 | ○1 | ○2 | ○3 | ○4 | ○5 | Much more |
| --- | --- | --- | --- | --- | --- | --- | --- | --- | --- | --- | --- | --- |

27. interact face to face with other international students who also stayed?

"0" means the frequency is the same as before.

| Much less | ○-5 | ○-4 | ○-3 | ○-2 | ○-1 | ○0 | ○1 | ○2 | ○3 | ○4 | ○5 | Much more |
| --- | --- | --- | --- | --- | --- | --- | --- | --- | --- | --- | --- | --- |

28. Since hearing about the Coronavirus, how would you rate your lifestyle?*

|  | Very different | Different | Rather different | Somewhat different, Somewhat similar | Rather similar | Similar | Very similar |
| --- | --- | --- | --- | --- | --- | --- | --- |
| Social environment (size of the community, pace of life, noise) | ○ | ○ | ○ | ○ | ○ | ○ | ○ |
| Living (hygiene, sleeping practices, how safe you feel) | ○ | ○ | ○ | ○ | ○ | ○ | ○ |
| Practicalities (getting around, using public transport, shopping) | ○ | ○ | ○ | ○ | ○ | ○ | ○ |
| Food and eating (what food is eaten, how food is eaten, time of meals) | ○ | ○ | ○ | ○ | ○ | ○ | ○ |
| Family life (how close family members are, how much time family spend together) | ○ | ○ | ○ | ○ | ○ | ○ | ○ |
| Social norms (how to behave in public, style of clothes) | ○ | ○ | ○ | ○ | ○ | ○ | ○ |
| Values and beliefs (what people think about religion and politics, what people think is right or wrong)? | ○ | ○ | ○ | ○ | ○ | ○ | ○ |
| People (how friendly people are, how stressed or relaxed people are, attitudes toward foreigners) | ○ | ○ | ○ | ○ | ○ | ○ | ○ |
| Friends (making friends, amount of social interaction, what people do to have fun and relax) | ○ | ○ | ○ | ○ | ○ | ○ | ○ |

29. I feel that too many demands are being made on me.

| ○Almost never |
| --- |
| ○Rarely |
| ○Occasionally |
| ○Usually |
| ○Always |

30. I find myself in situations of conflict.

| ○Almost never |
| --- |
| ○Rarely |
| ○Occasionally |
| ○Usually |
| ○Always |

31. I am under pressure from other people.

| ○Almost never |
| --- |
| ○Rarely |
| ○Occasionally |
| ○Usually |
| ○Always |

32. I feel criticized or judged.

| ○Almost never |
| --- |
| ○Rarely |
| ○Occasionally |
| ○Usually |
| ○Always |

33. I fear I may not manage to attain my goals.

| ○Almost never |
| --- |
| ○Rarely |
| ○Occasionally |
| ○Usually |
| ○Always |

34. I feel discouraged.

| ○Almost never |
| --- |
| ○Rarely |
| ○Occasionally |
| ○Usually |
| ○Always |

35. I am afraid for the future.

| ○Almost never |
| --- |
| ○Rarely |
| ○Occasionally |
| ○Usually |
| ○Always |

36. I feel under pressure from deadlines.

| ○Almost never |
| --- |
| ○Rarely |
| ○Occasionally |
| ○Usually |
| ○Always |

37. Thinking of your overall experience during the outbreak, please choose top 3 sources that have helped you smoothly get adapted.

Choose top 3 and mark with serial number.

| [ ]University faculty |
| --- |
| [ ]Embas/sie |
| [ ]Family |
| [ ]Friends |
| [ ]Other international students who also stayed |

38. Compared to normal life before, how do you feel about t**he overall support from university faculty and administration**?

| Not helpful | ○1 | ○2 | ○3 | ○4 | ○5 | ○6 | ○7 | ○8 | ○9 | ○10 | Very helpful |
| --- | --- | --- | --- | --- | --- | --- | --- | --- | --- | --- | --- |

39. Compared to normal life before, how do you feel about the overall support **from friends and family back home?**

| Not helpful | ○1 | ○2 | ○3 | ○4 | ○5 | ○6 | ○7 | ○8 | ○9 | ○10 | Very helpful |
| --- | --- | --- | --- | --- | --- | --- | --- | --- | --- | --- | --- |

40. Compared to normal life before, how do you feel about the overall support **from local Chinese friends?**

| Not helpful | ○1 | ○2 | ○3 | ○4 | ○5 | ○6 | ○7 | ○8 | ○9 | ○10 | Very helpful |
| --- | --- | --- | --- | --- | --- | --- | --- | --- | --- | --- | --- |

41. Compared to normal life before, how do you feel about the overall support **from other international students who also stayed in China?**

| Not helpful | ○1 | ○2 | ○3 | ○4 | ○5 | ○6 | ○7 | ○8 | ○9 | ○10 | Very helpful |
| --- | --- | --- | --- | --- | --- | --- | --- | --- | --- | --- | --- |

1. Note: In this survey, measurements used in the current study are highlighted, including No. 1 - 7，No. 17-18，No. 21，No. 25 - 26, No. 33 – 36, No. 38, No. 40 – 41. Others are used for other ongoing research projects and a MA thesis. [↑](#footnote-ref-1)
2. 2 Note: in the current study we used the measurements of contact online (with faculty, with friends, and with other international students) as sources of contact, as during the Wuhan lockdown students were not allowed to leave dormitory building and face to face contact was restrained. [↑](#footnote-ref-2)
